# Anticancer properties and pharmaceutical applications of ginsenoside compound K: A review

**DOI:** 10.1111/cbdd.13983

**Published:** 2021-11-25

**Authors:** Li Zhou, Zhong‐Kun Li, Cong‐Yuan Li, Yue‐Qin Liang, Fan Yang

**Affiliations:** ^1^ Department of Pharmacy Yan'an Hospital Affiliated to Kunming Medical University Kunming China; ^2^ Joint Surgery General Hospital of Tibetan Military Command Lhasa Lhasa China

**Keywords:** anticancer Effects, Compound K, Ginsenoside, molecular mechanisms, review

## Abstract

Ginsenoside compound K (CK) is the major intestinal bacterial metabolite of ginsenosides that exhibits anticancer potential in various cancer cells both in vitro and in vivo. The anticancer types, mechanisms, and effects of CK in the past decade have been summarized in this review. Briefly, CK exerts anticancer effects via multiple molecular mechanisms, including the inhibition of proliferation, invasion, and migration, the induction of apoptosis and autophagy, and anti‐angiogenesis. Some signaling pathways play a significant role in related processes, such as PI3K/Akt/mTOR, JNK/MAPK pathway, and reactive oxygen species (ROS). Moreover, the effects of CK combined with nanocarriers for anticancer efficiency are discussed in this review. Furthermore, we aimed to review the research progress of CK against cancer in the past decade, which might provide theoretical support and effective reference for further research on the medicinal value of small molecules, such as CK.

## Graphical abstract

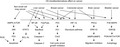

## 1 INTRODUCTION

Ginseng is the root and rhizome of *Panax ginseng* C.A. Meyer belonging to Araliaceae family (Kiefer & Pantuso, 2003). The name “Panax” is derived from the Greek word meaning “heal‐all;” ginseng with great medicinal value has been widely used in Asian countries for thousands of years (Kiefer & Pantuso, 2003; Wang, Anderson, et al., 2016). According to the earliest Chinese materia medica named *Shen*‐*nong's Herbal Classic* (Sun et al., 2016), ginseng is one of the most popular medicines for nourishing the body and replenishing vital energy without toxic side effects. Previous studies have shown that ginseng is regarded as the king of herb for its numerous benefits, such as improving cardiovascular health, enhancing immunity, inhibiting cancer metastasis (Cai & Yang, 2016), and protecting hepatorenal functions in traditional Chinese medicine (Hwang et al., 2016; Wong et al., 2015).

There are many components in ginseng, including ginsenosides, polysaccharides, polypeptides, and glycoconjugate compounds (Im & Nah, 2013). Among them, ginsenosides are the main components isolated from ginseng, which nearly 150 ginsenosides have been isolated and identified from roots, leaves, fruits, flower buds, processed items of ginseng, and other species (Sharma & Lee, 2020). Furthermore, it has been verified that ginsenosides are the marker compound for quality control and standardization of ginseng in official monographs (Qi et al., 2011). Ginsenosides are the main active ingredients of ginseng and are responsible for various pharmacological and biological effects of ginseng (Liu et al., 2020), which influence metabolism and immune, antioxidant, central nervous system, and cardiovascular systems, especially with respect to cancer treatment. As dammarane‐type triterpene glycosides, ginsenosides are divided into three types based on their chemical structures (Table 1: protopanaxadiols, protopanaxatriols, and oleanane (ginsenoside Ro). Protopanaxadiols, Rb1, Rb2, Rc, Rd, Rg3, and Rh2, consist of sugar moieties on the C‐3position. Protopanaxatriols, such as ginsenosides Re, Rf, Rg1, Rg2, and Rh1, have sugar moieties on the C‐6position. Since studies of parent ginsenoside activities in vitro may not accurately reflect their pharmacological effects in vivo conferred by metabolite effects, it is essential to investigate the effects of the metabolites based on enteric microbiota. Many macromolecular ginsenosides are chemically transformed in the gut upon consumption and absorbed as smaller metabolites. These metabolites are more bioavailable and pharmacologically active than their parent compounds (Wong et al., 2015).

**TABLE 1:** General classification of ginsenosides

Types	Ginsenoside
Protopanaxadiols	Ra1, Ra2, Ra3
	Rb1. Rb2, Rb3
	Rc
	Rd
	Rg3
	Rh2
	Compound K
Protopanaxatriols	Re
	Rf
	Rg1
	Rg2
	Rh1
Oleanane	Ro
	Rh3

**FIGURE 1:**
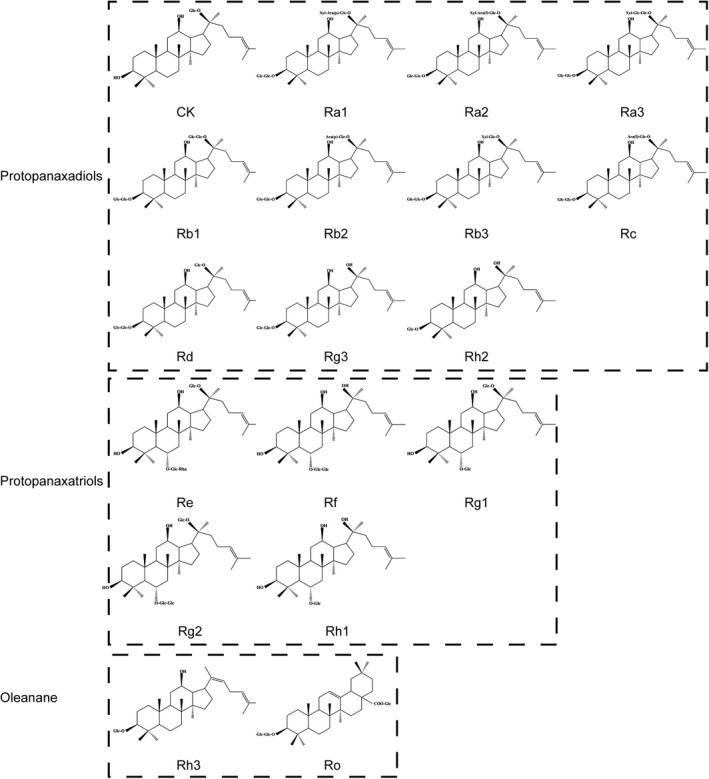
Chemical structure of classification of ginsenoids (Glc: Glucose, Ara:Arabinose, Xyl: Xylose, Rha: Rhamnose)

**FIGURE 2:**
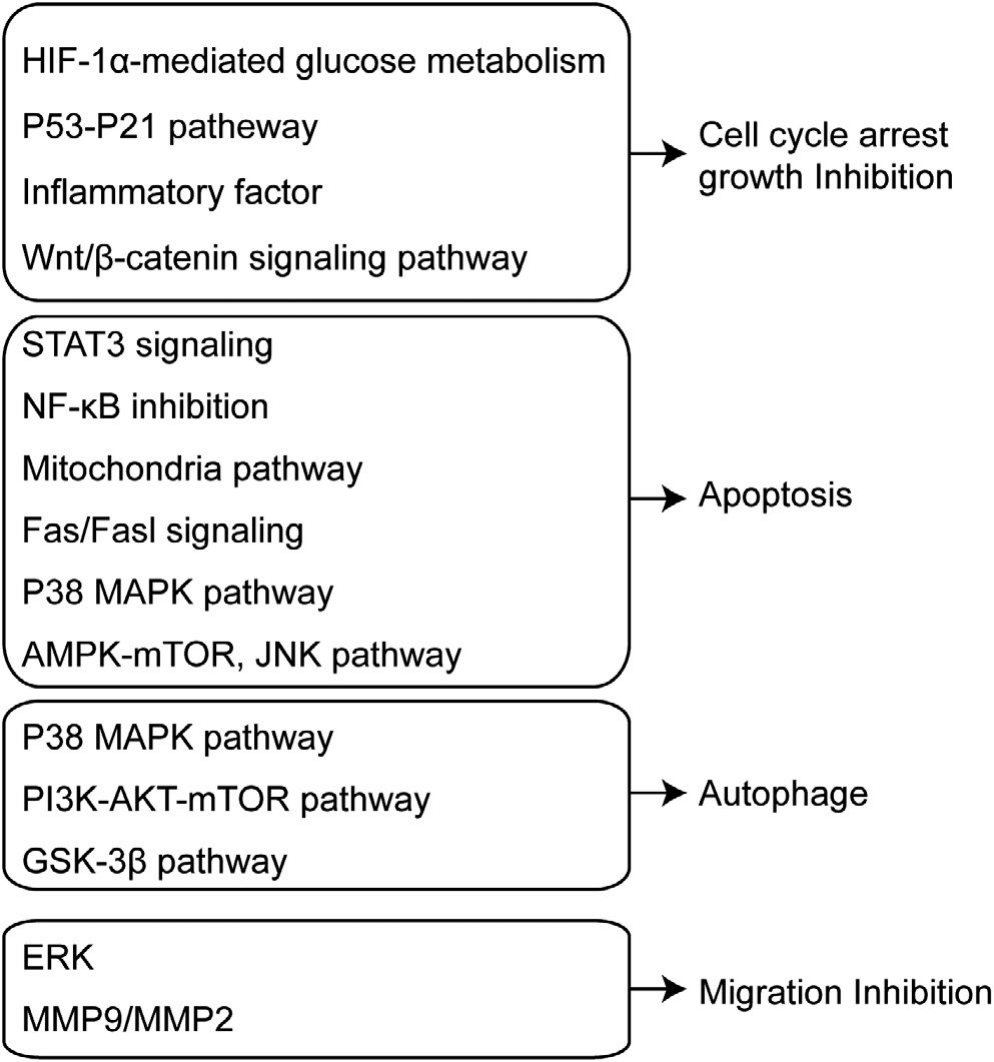
Schematic presentation of mechanisms for anticancer activities of CK

**FIGURE 3:**
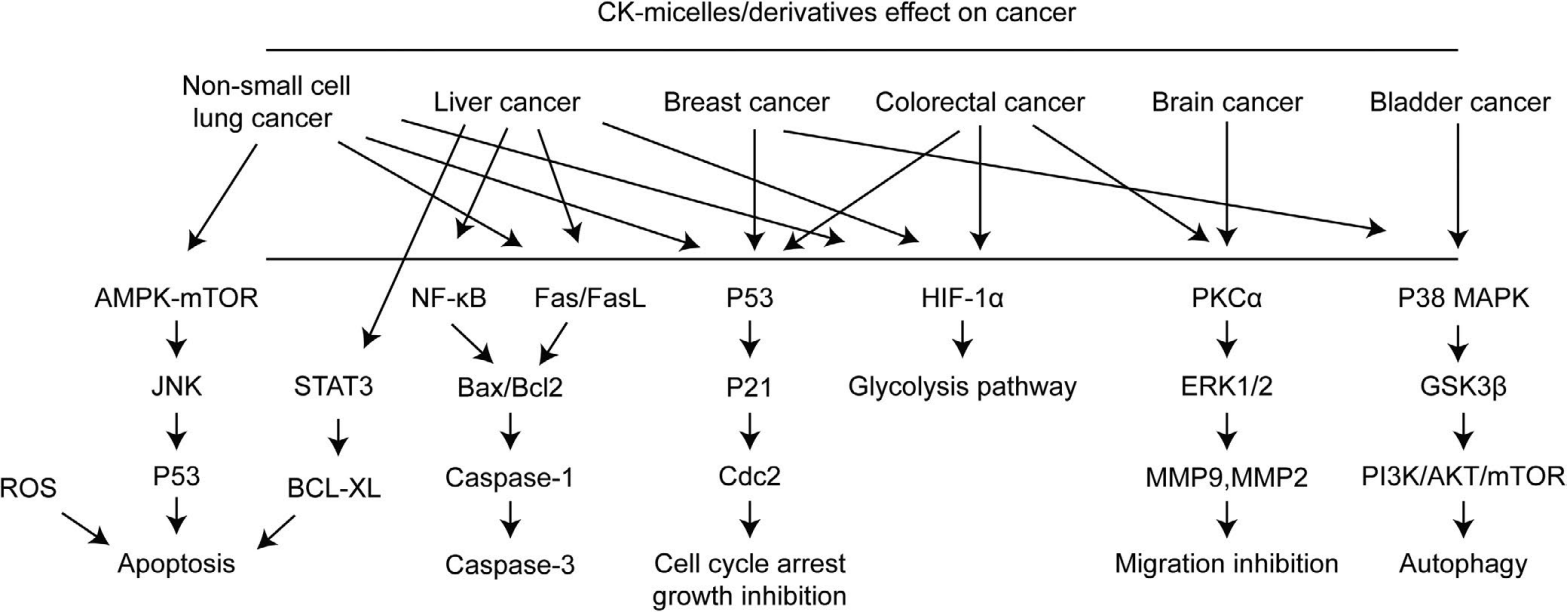
CK micelles/derivatives effect on cancer

Ginsenoside compound K (CK, 20‐O‐β‐(D‐glucopyranosyl)‐20(S)‐protopanaxadiol) is the major intestinal bacterial metabolite of ginsenosides (Figure 1), which is considered as the main functional component after ginseng or ginsenosides are consumed orally (Hasegawa, 2004). Specifically, CK is produced after major ginsenosides, Rb1, Rb2, and Rc, are deglycosylated by human gut bacteria. Furthermore, several studies have reported the bioconversion for CK using other methods, such as ginsenoside Rb1 converted into CK by *Leuconostoc citreum* LH1 isolated from kimchi (Quan et al., 2011). Over the decades, numerous effects of CK have been described (Sharma & Lee, 2020), among which the anticancer effect has been a hot spot of research. Interestingly, some anticancer effects of ginsenoside Rb1 are only detected in the metabolite form of CK but not the parent compound, further implying the importance of obtaining the active metabolites of CK by biotransformation and metabolism (Han et al., 2015; Li, Zhang et al., 2015; Wang et al., 2012; Yao et al., 2018). The present study aimed to summarize the research progress of the anticancer effect of CK and its major mechanisms (Figure 2, Figure 3). We also presented an arrangement and update about the effect of CK on cancer treatment over the last decade and summarized the mechanistic viewpoint in anticancer ginseng pharmacology. (Tables 2, 3)

**TABLE 2:** Summary of anticancer effects and mechanisms of CK

Cancer Types/Models	Material Type	Study Type	Effects	Mechanisms	Key Indicators	Reference
Non‐small cell lung cancer (NSCLC)	CK	In vitro	Changes in glucose metabolism, growth inhibition	HIF‐1α↓, GLUT1↓, HK II↓, PDK1↓, LDHA↓		Chen et al. (2019)
	CK	In vitro	Apoptosis	endoplasmic reticulum stress, accumulation of intracellular calcium, m‐calpain↑	IC50 of CK for A549cells and SK‐MES‐1 cells: 17.78 μm, 16.53 μm, respectively	Shin et al. (2018)
	CK	In vitro	Apoptosis, cell cycle arrest, autophagy	AMPK‐mTOR↑, JNK↑, LC3II↑, Beclin‐1↑, p62↓		Li et al. (2019)
	CK	In vitro	Enhances the efficacy of cisplatin, apoptosis, growth inhibition	p53↑(12–14 folds)	20 µm CK and 2.5 µm cisplatin alone: 4–8% apoptotic cells in H460 and A549 cells; co‐treatment: ~25%	Li, Zhou, et al. (2015)
	CK and parthenolide within tLyp‐1 liposomes	In vitro and in vivo	Apoptosis	ROS↑, mitochondrial apoptosis	ROS levels: CK(3.7%), parthenolide (5.8%); CK +parthenolide (24.6%); CK/parthenolide tLyp‐1 liposomes (28.7%)	Jin, Zhou, et al. (2018)
Liver cancer	CK	In vitro and in vivo	Apoptosis	endoplasmic reticulum stress, p‐STAT3↓, GRP78↑	IC50 of CK for HepG2, SMMC‐7721, Hep3B, and Huh7 cells were 40.45, 48.36, 45.55 and 41.93 μm	Zhang, Wang, et al. (2018)
	CK	In vitro	Apoptosis	targeting annexin A2, NF‐кB↓, caspase‐9↑, caspase‐3↑		Wang et al. (2019)
	CK	In vitro	Apoptosis	Fas↑, FasL↑, Bax/Bcl‐2↑, pro‐caspase‐9↓, pro‐caspase‐3↓, Akt phosphorylation↓	IC50 for chang‐liver and MHCC97‐H cells: 71.3±3.7 µm, 49.8±2.5 µm	Zheng et al. (2014)
	CK, Octyl ester of CK	In vitro and in vivo	Apoptosis	caspase‐dependent pathway: Bcl‐2↓, Bax↑, caspase‐3↑		Hou et al. (2018)
	CK	In vitro and in vivo, hypoxia	Proliferation inhibition	Bclaf1↓, HIF‐1α↓, HIF‐1α‐mediated glycolysis pathway↓	IC50 of CK in Bel‐7404 cells: 63.78, 38.52, and 28.88 μm at 24, 48, and 72 h, respectively, and those in Huh7 cells: 64.00, 38.54, and 28.31 μm, respectively	Zhang, Jiang, et al. (2020)
Breast cancer	CK	In vitro	Programmed necrosis	GSK3β phosphorylation↓, β‐catenin↓, cyclin D1↓	CK inhibited the proliferation of MCF‐7 at 50 and 70 µm: 21% and 59% inhibition at 24 h, and 35% and 88% at 48 h, respectively	Kwak et al. (2015)
	CK, CK combine with cisplatin	In vitro	Apoptosis, proliferation inhibition	N‐cadherin↓, vimentin↓, p‐Akt/Akt↓, fibronectin↓, E‐cadherin↑	proliferation inhibition rates in CK, DDP and CK +DDP groups at 48 h: 19.18 ± 2.25, 21.34 ± 2.84, and 43.37 ± 5.62, respectively	Zhang & Li, 2016)
	CK	In vitro	Apoptosis, inhibition of invasion, migration, and colony formation	AKT1↑, caspase‐7↑, caspase‐8↑, caspase‐9↑, Bcl‐2↓	CK (0–50 μm) increased apoptosis: from 3.91% to 19.57%. Live cells decreased from 90.7% to 63.06%	Choi et al. (2019)
Colorectal cancer	CK	In vitro	Apoptosis, cell cycle arrest	p53↑, caspase‐8↑, caspase‐9↑	CK at 50 μm completely inhibited cell growth (inhibition rate100%), increased caspase 3, 8, and 9 activities to 45.7 ± 3.1%, 77.6 ± 7.3%, and 68.6 ± 11.9%, respectively	Wang et al. (2012)
	CK	In vitro and in vivo	Apoptosis, cell cycle arrest	p53/p21↑, FoxO3a‐p27/p15↑, Smad3↑, cdc25A↓, CDK4/6↓, cyclin D1/3↓	CK (>30 μm) promoted both early and late stages of apoptosis in HCT‐116 cells. CK (30, 40, 50 μm) induced G1 cell cycle arrest: 43.6%, 60.3%, and 83.5%.	Zhang et al. (2013)
	CK	In vitro	Apoptosis	mitochondria‐dependent apoptotic pathway and MAPK pathway: ROS↑, cytochrome c↑, Bax↑, Bcl‐2↓, caspase‐9↑, caspase‐3↑, JNK↑	IC50 of CK in HT‐29 cells: 20 μg/ml	Lee et al. (2010)
	CK	In vitro	Autophagy, apoptosis	Atg5↑, Atg6↑, Atg7↑, ROS↑, JNK↑, Bax↑, Bcl‐2↓, caspase‐9↑, caspase‐3↑	IC50 of CK in HCT‐116 cells: 20 μg/ml	Kim et al. (2013)
	CK	In vitro	Apoptosis, cell cycle arrest	inflammatory‐associated colorectal cancer, interleukin‐8↓	After incubated with 20 µm CK for 6 and 12 h, the concentrations of IL‐8 were reduced from 180.33±4.32 to 92.56±3.46 and 70.05±2.32 pg/ml, respectively	Yao et al. (2018)
	CK	In vitro	Apoptosis, cell cycle arrest	RUNX3↑, p21↑	IC50 of CK in HT‐29 cells: 32 µm	Kang et al. (2013)
	CK	In vitro and in vivo	Proliferation inhibition	immunosuppresive effect of MDSCs↓, apoptotic MDSCs↑, Cox‐2↓, Arg‐1↓, IL‐1β↓, IL‐6↓, IL‐17↓		Wang, Li, et al. (2015)
	CK	In vitro	Apoptosis, autophagy	Mcl‐1↓, Bcl‐2↓, surviving↓, X‐linked inhibitor of apoptosis protein↓, Fas‐associated death domain‐like IL‐1‐converting enzyme‐inhibitory protein↓, Bax↑, tBid↑, cytochrome c↑, DR5↑, ROS↑, JNK↑	Results of a flow cytometry analysis for apoptosis: CK induced 21.15% apoptosis, TRAIL 15.22%, CK +TRAIL 98.05%	Chen et al. (2016)
	CK	In vitro	Apoptosis	caspase and p53‐dependent LGR5 inhibition: p53↑, LGR5↓, c‐Myc↓, procaspase3↓, Pin1↓, pro‐PARP↓, Bcl‐xL c‐Myc↓, Snail↓, Pin1↓	50 μm CK increased sub G1 population to 11.1% in HCT116p53+/+ cells more than to 7.08% in HCT116p53−/− cells	Pak et al. (2020)
	panax notoginseng saponins, CK	In vivo	Prevention of tumorigenesis and development	regulation on the microbiome balance		Chen et al. (2020)
Brain cancer	protopanaxadiol, CK	In vitro	Cell cycle arrest, inhibition of cell viability	N‐cadherin↓, integrin β1↓, phosphorylation of focal adhesion kinase↓, cyclin D1↓	IC50 of protopanaxadiol and CK: ~33 and ~30 μg/ml, respectively	Wanderi et al. (2016)
	CK	In vitro	Migration inhibition	p‐PKCα↓(94.67%), p‐ERK1/2↓(94.67%), MMP9↓(68%), MMP2↓(78%)	1 µm Ck showed the maximum inhibitory effect (95.3%) of SDF‐1‐stimulated migration of C6 cells	Kim et al. (2016)
	CK	In vitro	Apoptosis, inhibition of growth, migration and stemness	cyclin D1↓, cyclin D3↓, ROS↑, PI3K/Akt/mTOR↓, PARP↑, caspase‐9↑, caspase‐3↑, CD133↓, Nanog↓, Oct4↓, Sox2↓	CK (50 μm) induced G1 cell cycle arrest: arrested cells from 68.6 to 80.7% for U87MG cells and from 66.0 to 77.3% for U373MG cells	Lee et al. (2017)
	CK	In vitro and in vivo	Apoptosis, autophagic Inhibition	ROS↑, mitochondria damage↑	IC50 of CK for SK‐N‐BE(2), SH‐SY5Y, and SK‐N‐SH cells: 5, 7 and 15 μm, respectively	Oh et al. (2019)
Acute myeloid leukemia	CK combine with cytarabine	In vitro	Apoptosis, cell cycle arrest	mitochondrial dysfunction, DNA damage	IC50 of CK: 24.55 μm in U937 cells, 31.72 μm in THP‐1 cells, 20.11 μm in MCF‐7 cells, 36.34 μm in NCI‐H358 cells, 33.43 μm in C2C12 cells, 74.80 μm in HAEC cells	Qi et al. (2020)
Bladder cancer	CK	In vitro	Apoptosis	p38MAPK phosphorylation↑, ROS↑, cytochrome c↑, caspase‐9↑, caspase‐3↑, Bax/Bcl‐2↑,	5 μm was the minimal dose to inhibit the cell growth of T24: viable cells decreasing to 83.0%. 25 μm CK could reduce to 16.4%	Wang et al. (2013)
Nasopharyngeal carcinoma	CK	In vitro and in vivo	Apoptosis	mitochondrial pathway	IC50 of 20(S)‐Rh2, CK, PD, and PPD on HK‐1 cells: 12, 11.5, 8, and 7 μm, respectively	Law et al. (2014)
Ovarian carcinoma	Rb1, CK	In vitro and in vivo	growth inhibition	inhibition of Wnt/β‐catenin signaling and epithelial‐to‐mesenchymal transition	LC50: 250 nm for Rb1 and 100 nm CK in SKOV‐3 cells, 230 nm for Rb1 and 125 nm for CK in HEYA8 cells, respectively	Deng et al. (2017)
Renal cell carcinoma	CK	In vitro	Apoptosis, inhibition of growth, invasion and migration	ROS↑, lncRNA THOR↓, caspase‐9↑, caspase‐3↑		Chen et al. (2021)

**TABLE 3:** Anticancer effects of CK micelles/derivatives

Material Type	Study Type	Model	Effects (compared to CK)	Key Indicators	References
GC‐CK conjugate	In vitro	HT29, HepG2, and HT22 cells	solubility↑, targeted delivery↑, cytotoxicity↑	1. The stability of GC–CK4 conjugates was maintained for 8 days. 2. GC–CK exhibited significantly higher or similar cytotoxicity compared with CK in HT29 and HepG2 cells, and slightly lower toxicity than CK on HT22 cells	Mathiyalagan et al. (2014)
DCY51T‐AuCKNps	In vitro	A549 cells, HT29 cells	cytotoxicity↑, apoptosis↑	1. DCY51T‐AuCKNps showed preferential cytotoxicity against A549 and HT29 cells compared to free CK. 2. DCY51T‐AuCKNps increased apoptosis in cancer cells compared to RAW264.7 cells	Kim et al. (2019)
CK AP / TPGS	In vitro and in vivo	A549 cells, A549 lung cancer xenograft mouse model	targeted delivery↑, proliferation↓, apoptosis↑	1. IC_50_ of free CK and CK mixed micelles at 24 h: 16.11±1.23, 10.29±1.17 μg/ml, respectively. 2. Apoptosis index: free CK 17.28%±2.25%, CK mixed micelles 45%±5.25%.	Zhang et al. (2017)
CK PC / DP	In vitro and in vivo	A549 cells, A549 lung cancer xenograft mouse model	solubility↑, permeability↑, apoptosis↑, anti‐invasion↑	1. Solubility increased almost 66‐fold: CK 33.15 ± 3.82μg/ml, CK PC/DP micelles 2,215.67 ± 166.39 μg/ml. 2. IC50 of free CK and CK PC/DP micelles at 24 h: 18.31 and 12.15 μg/ml, respectively. 3. Percentage of cells in G1 phase: free CK 31.54% ±2.48%, CK PC/DP micelles 39.27% ±4.39%.	Jin, Yang, et al. (2018)
CK‐TPGS/PEG‐PCL	In vitro and in vivo	A549 cells, PC‐9 cells, A549 lung cancer xenograft mouse model	solubility↑, apoptosis↑, anti‐invasion↑	1. IC_50_ of CK and CK‐TPGS/PEG‐PCL in A549 cells: 21.97±1.50 and 25.43±2.18 μg/ml, respectively. 2. IC_50_ of CK and CK‐TPGS/PEG‐PCL in PC‐9 cells: 14.46±1.24 and 18.35±1.90 μg/ml, respectively.	Yang et al. (2017)
CK‐NPs	In vitro	HepG2 cells	solubility↑, cytotoxicity↑, apoptosis↑	1. IC_50_ of CK and CK‐NPs in HepG2 cells: 23.33 and 16.58 μg/ml, respectively. 2. Percentages of apoptotic cells treated with CK and CK‐NPs: 39.02 ± 0.42% and 47.57 ± 1.65%, respectively.	Zhang, Zhang, et al. (2018)
APD‐CK	In vitro	HepG2 cells, Huh‐7 cells	cytotoxicity↑, apoptosis↑	IC_50_ of CK and APD‐CK micelles: after 24 h, 33.62 and 19.35 µg/ml, respectively. after 48 h, 28.19 and 16.32 µg/ml, respectively.	Zhang, Jiang, et al. (2020)
CK‐OCMC Nps	In vitro	PC3 cells	solubility and stability, permeability, cytotoxicity↑, apoptosis↑	1. Cell viability after 24 h of incubation with CK–OCMC Nps (30 μg/ml) and CK (30 μg/ml): 12.11 ± 5.33% and 29.28 ± 4.84%, respectively. 2. CK–OCMC enhanced the levels of caspase‐3 and caspase‐9 by 29.93% and 20.78% compared with free CK.	Zhang et al. (2021)

Several articles with two keywords in their “Title/Abstracts” were retrieved from PubMed from 2010 to date: ginsengplus cancer (735 papers), ginsenoside plus cancer (583 papers), or compound K plus cancer (80 papers). Compared to ginseng and ginsenoside, the number of papers on compound Kand cancer has not increased markedly.

## 2 ANTICANCER ACTIVITIES OF CK

### 2.1 Lung cancer

Non‐small cell lung cancer (NSCLC) is the leading cause of cancer‐related deaths. Among recent findings, a study investigated the suppressing effect of CK on NSCLC cell growth via hypoxia‐inducible factor‐1α (HIF‐1α) mediated glucose metabolism alteration. Briefly, CK inhibited the cell viability of NSCLC cells, decreased the glucose uptake and lactate secretion under normoxic and hypoxic conditions, and inhibited the expression of HIF‐1α and its downstream gene *GLUT1*. On the contrary, the over‐expression of HIF‐1α elevated the metabolic reactions and partly attenuated the inhibitory role of compound K on NSCLC cell growth (Chen et al., 2019). Another study elucidated that CK induced the apoptosis of A549 and SK‐MES‐1 human lung cancer cells, in which cell survival and intracellular Ca^2+^ homeostasis during ER stress in human lung cancer cells were major inducing factors (Shin et al., 2018). In addition, it has been suggested that CK suppressed the proliferation and promoted apoptosis and autophagy in A549 and H1975 cells by activating AMP‐activated protein kinase/mammalian target of rapamycin (AMPK‐mTOR) and c‐Jun N‐terminal kinase (JNK) signaling pathways (Li et al., 2019). Many researchers focused on the synergistic antitumor efficacy of CK and other medications. For example, a study provided the first evidence that CK increases the efficacy of cisplatin in lung cancer by enhancing the cisplatin‐induced p53 expression and activity (Li, Zhou, et al., 2015), which indicated that the combined CK and cisplatin had better effects than either molecule alone. Another study aimed to prepare CK‐loaded liposomes modified with D‐alpha‐tocopheryl polyethylene glycol succinate (TPGS) for increasing the solubility and targeting the ability of CK and also confirmed that CK‐liposomes significantly improved the efficacy of CK against NSCLC, as assessed by in vitro and in vivo evaluation (Yang et al., 2016). Furthermore, a study demonstrated that combined parthenolide and CK within tLyp‐1 liposomes effectively induce mitochondria‐mediated lung cancer apoptosis (Jin, Zhou, et al., 2018).

### 2.2 Liver cancer

A study explored the effects of CK on liver cancer in HepG2 cells, SMMC‐7721 cells, and mice‐bearing human hepatocellular carcinoma (HCC) xenografts, indicating that CK decreased the DNA‐binding capacity of STAT3 in HepG2 and SMMC‐7721 cells. Silencing STAT3 with CRISPR/Cas9 technology enhances CK‐induced ERS and apoptosis. Thus, CK induced ERS and apoptosis by inhibiting p‐STAT3 in human liver cancer cells (Zhang, Wang, et al., 2018). Another study demonstrated the molecular targets for anticancer activity of CK using HepG2 cells and confirmed that CK inhibits nuclear factor‐kappa B (NF‐kB) mainly by targeting Annexin A2 (Wang et al., 2019), thereby exerting cytotoxic activity. In addition, a previous study showed that CK inhibits cell proliferation and induces apoptosis in human hepatocellular carcinoma cell line MHCC97‐H cells through Fas‐ and mitochondria‐mediated caspase‐dependent pathways (Zheng et al., 2014). Subsequently, CK increased the expression of Fas, FasL, and Bax/Bcl‐2 ratio, decreased the expression of pro‐caspase‐9 and pro‐caspase‐3, and inhibited Akt phosphorylation.

A study reported a synthetic method of CK‐O, a mono‐octylester of CK, to evaluate its anticancer effects. Interestingly, CK‐O showed cytotoxicity and pro‐apoptosis effects in murine H22 cells both in vitro and in vivo in a dose‐dependent manner (Hou et al., 2018). Recently, some studies focused on CK‐mediated inhibition of the proliferation of human liver cancer cells by regulating HIF‐1α‐mediated glycolysis. It was demonstrated that CK reduces the expression of Bcl‐2‐associated transcription factor 1 in hypoxic liver cancer cells and inhibits the HIF‐1α‐mediated glycolysis pathway, ultimately inhibiting cell proliferation (Zhang, Zhang et al., 2020).

### 2.3 Breast cancer

Breast cancer is a malignant disease and the second leading cause of cancer‐related deaths in women worldwide. A large number of studies and treatment options for breast cancer have been developed. The results of an early study devoted to directly measuring the cellular behavior of ginsenosides, such as Rg3, Rg5, Rh2, and CK on cancer cells, indicated that the different anticancer activities could be attributed to the selective uptake of ginsenosides based on various chemical structures (Ha et al., 2010). Another study reported that CK inhibited the proliferation of MCF‐7 breast cancer cells. In addition, CK induced programmed necrosis, but not apoptosis, through GSK3β signaling pathway in MCF‐7 cells (Kwak et al., 2015). Additionally, CK inhibited the proliferation and epithelial‐to‐mesenchymal transition and induced apoptosis in MCF‐7 cells via the PI3K/Akt pathway. The combination of CK with cisplatin is effective against breast cancer (Zhang & Li, 2016). Also, CK inhibited SKBR3 and MDA‐MB‐231 cell viability, invasion, migration, and colony formation and induced apoptosis of these breast cancer cells by regulating the AKT1 activity (Choi et al., 2019).

### 2.4 Colorectal cancer

As mentioned above, CK, but not its parent ginsenoside Rb1, showed significant antiproliferative and pro‐apoptotic effects in HCT‐116 and SW‐480 colorectal cancer cells, suggesting potential chemopreventive activities of CK in human colorectal cancer (Wang et al., 2012). Another study showed the anticancer effects of CK on colorectal cancer in vivo and in vitro, such as antiproliferation, pro‐apoptosis, and changes in the cell cycle distribution. These effects could be attributed to the up‐regulation of p53/p21, FoxO3a‐p27/p15, and Smad3 and the down‐regulation of cdc25A, CDK4/6, and cyclin D1/3, which indicated that CK inhibited colorectal cancer growth via multiple pathways including p53‐p21 interactions (Zhang et al., 2013). A previous study elucidated the cytotoxic mechanism of CK‐induced apoptosis of HT‐29 human colon cancer cells, which demonstrated that CK‐mediated generation of reactive oxygen species (ROS) resulted in apoptosis by modulating the mitochondria‐dependent apoptotic and JNK/p38mitogen‐activated protein kinase (MAPK) pathways (Lee et al., 2010). CK also induced autophagy and apoptosis of human HCT‐116 colon cancer cells via the generation of ROS and activation of JNK (Kim et al., 2013). Among recent findings, a study compared the effects of ginsenoside Rb1 and CK on colorectal cancer using the HCT‐116 and HT‐19 human colorectal cancer cell lines by MTS assay, flow cytometry, and ELISA. The results showed that CK is the major intestinal microbiome metabolite of Rb1 and exhibits strong antiproliferative effects. Moreover, CK exerted significant anti‐inflammatory effects at low concentrations and induced cell apoptosis, while Rb1 did not have any distinct effects (Yao et al., 2018). In another study, HT‐29 human colon cancer cells were treated with CK (Kang et al., 2013), resulting in the inhibition of colorectal cancer cell growth and induction of apoptosis by inhibiting histone deacetylase activity and increasing the expression of RUNX3 and the downstream target p21. In addition, CK increased apoptosis and suppressed the immunosuppresive effect and pro‐inflammatory cytokine production of myeloid‐derived suppressor cells (MDSCs) to inhibit colorectal cancer cell proliferation both in vitro and in mice‐bearing CT26 tumor xenograft (Wang, Li, et al., 2015). Another article reported that CK potentiated tumor necrosis factor‐related apoptosis‐inducing ligand (TRAIL)‐induced apoptosis in human colon cancer cells via autophagy‐dependent and ‐independent DR5 up‐regulation (Chen et al., 2016). A recent study revealed that leucine‐rich repeat containing G protein‐coupled receptor 5 (LGR5) was over‐expressed in colorectal cancers. Then, CK inhibited the expression of caspase and p53‐dependent LGR5, which induced apoptosis in colorectal cancer cells. Moreover, it was proved that CK had synergistic antitumor potential with 5‐FU or doxorubicin (Pak et al., 2020). Another study confirmed that *Panax notoginseng* saponins and its main bio‐transformed metabolite CK prevented the development of colorectal cancer associated with colitis with respect to regulating intestinal flora (Chen et al., 2020).

### 2.5 Brain cancer

Glioblastoma is the most aggressive and malignant form of primary brain cancer. A preliminary study demonstrated that CK reduces the viability of glioblastoma cells U251‐MG and U87‐MG in a dose‐ and time‐dependent manner (Wanderi et al., 2016). Kim et al. reported the inhibitory effect of CK on stromal cell‐derived growth factor 1 (SDF‐1) pathway‐induced migration of C6 glioma cells, which suggested that CK inhibits C6 glioma cell migration by regulating the downstream signaling molecules, including protein kinase C (PKC)α, extracellular signal‐regulated kinase (ERK), and matrix metallopeptidases (MMP) (Kim et al., 2016). Furthermore, a study investigated the anticancer effect of CK against glioblastoma cells, U87MG and U373MG. The results showed that CK significantly inhibited growth, metastatic ability, and stemness of glioblastoma cells through PI3K/Akt/mTOR signaling pathway (Lee et al., 2017).

Among studies about neuroblastoma, Oh et al. demonstrated the effects of ginsenoside CK on human neuroblastoma cells in vitro and in vivo. These effects could be attributed to proliferation inhibition, ROS‐mediated apoptosis, autophagosome accumulation, and autophagic flux inhibition. The present study proved that chloroquine promotes CK‐induced apoptosis, mitochondrial ROS induction, and mitochondria damage (Lee et al., 2017; Oh et al., 2019).

### 2.6 Other cancers

Pediatric acute myeloid leukemia (AML) is a heterogeneous disease. A study investigated the effects of CK on clinically relevant pediatric AML cell lines and confirmed that CK inhibits cell growth and induces cell apoptosis. The effects were related to the suppression of DNA synthesis (Chen et al., 2013) and DNA double‐strand breaks. A new study discovered that CK combined with cytarabine synergistically induces DNA damage in AML cells, which is appropriate because CK significantly reduced the resistance to cytarabine and improved AML treatment (Qi et al., 2020).

Wang et al. investigated bladder cancer and revealed that CK decreases the cell number and induces apoptosis of bladder cancer T24 cells, whose mechanism was partially due to ROS generation and p38‐MAPK activation (Wang et al., 2013). In addition, a study showed that CK significantly induced apoptosis of the nasopharyngeal carcinoma cell line HK‐1 and inhibited the HK‐1 xenograft tumor growth, which was related to the activation of apoptosis‐inducing factors (Law et al., 2014).

To ease chemoresistance during cancer treatment, researchers showed for the first time that ginsenoside Rb1 and its metabolite CK could target chemotherapy‐resistant ovarian cancer stem cells by simultaneous inhibition of epithelial‐to‐mesenchymal transition and Wnt/β‐catenin signaling (Deng, Wong, Lai, & Wong, 2017). In addition, a recent study found that CK significantly inhibited the proliferation, migration, and invasion of renal cell carcinoma cells and induced caspase‐dependent apoptosis. These findings were related to the regulation of ROS and lncRNA testis‐associated oncogenesis (Chen et al., 2021).

## 3 CK MICELLES/DERIVATIVES EFFECT ON CANCER

Numerous studies have described the anticancer effects of CK. However, there are still limited applications in clinical settings because of low solubility and poor absorption of CK. To overcome these shortcomings, a previous study conjugated CK to the backbone of hydrophilic glycol chitosan (GC) through an acid‐labile linkage. The in vitro experiments revealed that GC‐CK conjugate significantly enhanced water solubility and targeted delivery of CK and also exhibited higher cytotoxicity than CK in HT29, HepG2, and HT22 cell lines (Mathiyalagan et al., 2014). Furthermore, researchers carried out the rapid green synthesis of silver and gold nanoparticles (NPs) using dendropanax that could be used as carriers to carry CK for cancer therapy on account of their lack of normal cytotoxicity (Wang, Mathiyalagan, et al., 2016). Further study showed that CK loaded by gold NPs could act as a potent photothermal therapy agent for treating cancers (Kim et al., 2019).

In addition, CK ascorbyl palmitate (AP)/d‐α‐tocopheryl polyethylene glycol 1000 succinate monoester (TPGS) mixed micelles (CK AP/TPGS) were prepared. Then, it was confirmed that CK AP/TPGS mixed micelles enhanced tumor targeting and antitumor effects, such as reducing proliferation, inhibiting migration, and promoting apoptosis in an in vitro A549 cell model and A549 lung cancer xenograft mouse model (Zhang et al., 2017).

Moreover, a study applied the micellar system based on phosphatidylcholine (PC) and 1,2‐distearoyl‐sn‐glycero‐3‐phosphoethanolamine polyethylene glycol 2000 (DP) and showed that CK encapsulated in PC/DP mixed micelles had improved solubility, permeability, and retention effects (Jin, Yang, et al., 2018). Similarly, it was confirmed that targeted delivery of CK significantly enhanced the treatment of lung cancer (Yang et al., 2017). In another study, chitosan NPs loaded with CK (CK‐NPs) were prepared as a delivery system, which further revealed that CK‐NPs improved the water solubility of CK, enhanced the cytotoxicity and apoptosis of HepG2 cells with an IC50 value of 16.58 μg/ml, and promoted cellular uptake in vitro (Zhang, Wang, et al., 2018).

Recently, A54 peptide was used to fabricate CK‐loaded micelles (APD‐CK) for liver targeting. The results showed that APD‐CK micelles enhanced the cellular uptake of micelles and promoted cell apoptosis in vitro in HepG2 and Huh‐7 cells (Zhang, Jiang, et al., 2020).

A new study discovered that CK‐loaded ionically cross‐linked carboxymethyl chitosan‐calcium nanoparticles enhance the water solubility and permeability, thereby increasing the cytotoxicity and cellular uptake of CK toward prostate cancer cells (Zhang et al., 2021).

## 4 OTHER EFFECTS OF CK

As described previously, CK has been currently reported as a promising and effective agent in anticancer, anti‐inflammation, anti‐angiogenesis, antioxidant, and neuroprotection. An earlier study demonstrated that CK exhibited anti‐angiogenic activity in human umbilical vein endothelial cells by inhibiting p38 MAPK and AKT with the potential for a cancer chemopreventive agent (Jeong et al., 2010). Another study suggested that ginsenoside Rb1 and CK had an antidepressant‐like effect that may be regulated by 5‐HT(2A) receptors(Yamada et al., 2011). Park et al. confirmed that CK inhibited platelet‐derived growth factor (PDGF)‐BB‐induced vascular smooth muscle cell proliferation and migration via G1 arrest and significantly attenuated neointima formation after arterial injury by building a rat carotid artery injury model (Park et al., 2013). A study showed that CK promotes the recovery of dextran sulfate sodium‐induced colitis and inhibits the inflammatory responses by suppressing NF‐κB activation. Moreover, CK reduces intestinal inflammation by inhibiting the production of the pro‐inflammatory cytokines (Li et al., 2014). Another study verified the anti‐angiogenic ability of CK against sphingosine 1‐phosphate‐induced cell migration by regulating the level of sphingosine kinase 1 in human umbilical vein endothelial cells (Shin et al., 2014). Reportedly, CK modulates the immune responses during pathogenic bacterial and viral infections, which exhibits beneficial effects in infectious diseases (Nguyen & Nguyen, 2019). Recently, a study proved that CK clearly improves skin barrier function in an atopic dermatitis‐like model by increasing expression of the serine protease inhibitor Kazal type‐5 (Park et al., 2020). A new study verified that CK protects melanocytes against oxidative stress through adjusting redox balance between glutathione and oxidized glutathione, and also alleviates leukoderma in guinea pigs. Therefore, CK may be a good candidate for preventing many kinds of skin diseases (Tang et al., 2021). In addition, a study recruited six healthy adults who regularly consumed different diets, including ginseng, and verified that subjects on a Western diet had much higher CK levels than those on an Asian diet. Therefore, people on a Western diet should obtain better cancer prevention effects with ginseng intake compared to those on an Asian diet (Wan et al., 2017).

Consistent with the reports of other studies, our multifaceted studies in recent years have also confirmed that CK has anti‐inflammatory, immunoregulatory, and neuroprotective effects. Also, it significantly attenuates the development of atherosclerosis by activating the reverse cholesterol transport pathway, reducing systemic inflammatory cytokines, and inhibiting local inflammasome activity (Zhou et al., 2016). CK derivatives modified with short‐chain fatty acids showed similar or better biological activity than CK (Huang et al., 2017). Conversely, CK promotes neurogenesis and proliferation, reduces apoptosis of neural stem cells after intracerebral hemorrhage and improves the neurological deficit (Zhou et al., 2020).

## 5 CONCLUSIONS AND PERSPECTIVES

A large number of studies suggested that the biological properties of CK are anti‐inflammation, anti‐oxidation, anti‐angiogenesis (Wang, Cai, et al., 2015), antiproliferation of tumor cells, and induction of apoptosis. Several studies reviewed the anticancer effects of CK but did not delve into the underlying mechanisms with respect to cancer. In recent years, no relevant detailed review has described the anticancer effect of CK. Therefore, we summarized and updated the studies on the anticancer effects of CK in the last decade to identify the most effective targets and mechanisms, which might provide a theoretical basis and evidence for transformation and application in the near future.

As presented above, with the potential of anticancer effects at the level of transcription, translation, and protein expression through a variety of mechanisms of action, CK proved to be more potent and valuable than its precursors. However, current studies mainly focused on the anticancer properties of CK in vitro and in animal models, whose effects might not be applicable to humans. Furthermore, several studies attempt to produce more CK or modify its structure using various preparation methods. For example, the combinations of CK with tumor‐targeting carriers improved the water solubility for enhanced cancer treatment. Several factors, such as different precursors, chemical carriers, action time, and preparation processes, can impact the harvest and the pharmacological effects of CK. Therefore, further studies are required to elucidate the influence of these variables and further illustrate the role of CK in anticancer effects. In addition, chemoresistance is one of the most difficult clinical problems compromising the successful treatment of cancer. Notably, some studies focused on the combination of CK and antitumor agents or the eradication of cancer stem/tumor‐initiating cells. Another study confirmed that CK inhibits the self‐renewal of cancer stem/tumor‐initiating cells derived from ovarian carcinoma patients and in the xenograft tumor model (Deng et al., 2017). Moreover, Hwan‐Suck et al. investigated 12 ginsenosides for their ability to block programmed death receptor‐1/programmed death ligand‐1 (PD‐1/PD‐L1) interactions using competitive ELISA, among which Rg3 and CK exhibited the highest inhibitory effects for PD‐1/PD‐L1 interactions. Consequently, CK and its precursor compounds might be crucial for the overall immuno‐oncological strategy (Yim et al., 2020). Intriguingly, a study on the role of cytochrome P450s (CYPs) in CK metabolism suggested that CK is a putative substrate and inhibitor for CYP2C9 and CYP3A4. Therefore, patients should be cautious while using CK in combination with therapeutic drugs that were definite substrates of CYP2C9 and CYP3A4 (Xiao et al., 2016).

Taken together, the studies provided baseline evidence and theoretical results for exploring the value of Chinese medicinal materials and their small molecule compounds, which have practical significance. Future studies should focus on the transformation from animal tumor model to human clinical treatment to gain the complete medicinal value of Chinese medicine and its small molecules.

## CONFLICT OF INTEREST

The authors declare no conflict of interest.
